# Use of and Factors Associated With the Use of Mobility Assistive Devices Among Older Adults in Low-Income Housing

**DOI:** 10.1093/geroni/igaf122.3367

**Published:** 2025-12-31

**Authors:** Stephanie McCorvey, Barbara Resnick, Nicole Brandt, Jennifer Klinedinst, Sarah Holmes

**Affiliations:** University of Maryland at Baltimore, Baltimore, Maryland, United States; University of Maryland, Baltimore, Maryland, United States; UMB School of Pharmacy, 20 N. Pine St. Baltimore, Maryland, United States; UMB SON, 655 W. Lombard Street, Baltimore, Maryland, United States; University of Maryland School of Nursing, Baltimore, Maryland, United States

## Abstract

There is a growing number of older adults in low-income housing communities that live with disabilities. Many of these individuals use mobility assistive devices (MADs) including walkers, canes, or wheelchairs, which they obtain from others, purchase or are ordered by a health care provider. This study explored the use of MADs among residents in low-income housing and factors associated with MAD use. It was hypothesized that the majority of individuals would use a MAD; and that perceived health status, cognition, exercise, unsteadiness, fear of falling, a fall in the past year, and fall-related injury, dizziness, and musculoskeletal or neurological disease would be associated with use of an MAD. Data were obtained from older adults (N = 220) who participated in an Annual Wellness Visit as part of an interdisciplinary wellness clinic. The mean age of participants was 79.5 (SD = 8.5), the majority was female (77%), Black (69%) and non-Hispanic (80%). Over half (53%) used a MAD. Worrying about falling (Wald=5.4, p =.02), feeling unsteady (Wald=19.7, p<.001), and health status (Wald 4.3, p=.04) were all significantly associated with MAD use. Those who worried were 2.9 times more likely to use a MAD, those who felt unsteady were 5.8 times more likely to use an MAD, and those who reported worse health status were 0.44 times less likely to use a MAD. Individuals seem to self-select use of a MAD based on concerns around falls. Future research should focus on the appropriateness of these devices at the individual level for safety and cost effectiveness.

